# Implementing a Peer Advocate Mental Health Digital Intervention Program for Ohio Youth: Descriptive Pilot Study

**DOI:** 10.2196/24605

**Published:** 2021-04-23

**Authors:** Tashuna Albritton, Kelsey Lynett Ford, Kira Elsbernd, Melodie Santodomingo, Ivan Juzang, Pam Weddington, Sheana Bull

**Affiliations:** 1 CUNY School of Medicine City College of New York New York, NY United States; 2 Colorado School of Public Health University of Colorado Aurora, CO United States; 3 MEE Productions Inc Philadelphia, PA United States

**Keywords:** mental health, adolescent, digital health, suicide prevention, social support, youth

## Abstract

**Background:**

In the United States, millions of adolescents report poor mental health, where 1 in 5 teenagers considers suicide. Reducing stigma and fostering peer support remains critical for positive mental health interventions and programs. Increasingly, digital mental health tools have emerged with great promise, leveraging social networks. Despite the potential, limited understanding of such comprehensive programs and their implementation exist.

**Objective:**

The objective of this study investigates a piloted digital mental health training program (Be Present) for youth, specifically describing the impact on youth behavioral outcomes and user engagement and identifying high-risk youth in the early phases of prevention.

**Methods:**

Eligibility included Ohio residents (aged 14 to 22 years) to be enrolled as either a Friend or a Peer Advocate. From May 1 to June 1, 2019, participants completed the Advocate training course, taking pretest and posttest surveys. Single-arm descriptive analyses measured youth outcomes (self-efficacy, intentions, behaviors, social support, knowledge, and sources of strength) and engagement and assessed risk based on survey responses.

**Results:**

A total of 65 adolescents participated, with 54 completing both pretest and posttest surveys. The majority of participants included non-Hispanic White females. Findings illustrated a significant increase in self-report of referrals for mental health services as well as in perceptions that youth had of experiencing social support; however, no significant differences were found for measures of self-efficacy, knowledge, and sources of strength between pretest and posttest surveys. Roughly two-thirds of the participants completed all of the Advocate training modules, and we observed a gradual decline in engagement. Most respondents who received escalated high-risk response messages identified as female.

**Conclusions:**

The pilot presented promise for implementing a digital mental health program focused on peer support, specifically observing reported youth behavioral outcomes and user engagement and identifying high-risk youth. Various limitations exist given the small nonrepresentative sample and lack of control group. All findings should be considered preliminary to a larger trial and underscore the feasibility of delivering online training programs to bolster adolescent mental health. Such formative evaluation proved critical for future implementation and research, offering opportunity for substantial improvements for real-world digital mental health programs.

## Introduction

### Background

In the United States every year, 1 in 5 teenagers considers suicide, and approximately 1 million teenagers have reported attempting suicide [1]. Suicide is the third leading cause of death for those aged 10 to 19 years [2]. People who considered suicide later reported the teenage years as the initial onset of suicidal ideation, making this time critical for a positive intervention [3]. Ohio’s youth, defined as those aged 15 to 24 years, had a suicide rate of 11.27 per 100,000, which is comparable to the United States average for youth of the same age, at 11.39 per 100,000 [4]. Addressing mental illness and stigma associated with mental illness and equipping teens with skills to handle stressors associated with this period of life is critical for an intervention that targets suicide prevention.

Peer support is critical for any intervention targeting teenagers; this is especially true in mental health interventions. Youth who are suicidal are more likely to talk to other youth about being suicidal than to adults [5]. Understanding this peer support in teens can be coupled with mental health education to both raise awareness and challenge existing stigma associated with mental illness [6]. Social support has shown direct effects on mental health outcomes, particularly with peers [7].

Online social media networks may then increase the perceived social support along with increasing the information spread of user-generated content [8]. These online social networks can also be used to reach large numbers of teens as a platform for prevention and education interventions; 83% of young adults have reported using at least one social media site [9]. Frequent use of the internet, for teens, has been reported to increase communications both with family and other social support, beyond social media into face-to-face relationships [10]. Despite the potential of social networks and ability to support mental health initiatives with technology, limited understanding exists on how to implement these initiatives in the real world [11]. The objective of this study investigates a pilot digital mental health training program for youth, specifically describing impact on youth behavioral outcomes and user engagement and identifying high-risk youth in the early phases of prevention. Given the importance of this prevention program, it was equally important to conduct a program evaluation to assess the impact of the content on youth outcomes, its effectiveness in maintaining youth interest as a novel digital suicide prevention program, and its effect on youth most vulnerable to suicide.

### Context

In response to ongoing concerns related to youth mental health, the Ohio Department of Mental Health and Addiction Services (Ohio MHAS) and the Motivational Educational Entertainment Productions Inc (MEE) team created Be Present, a digital e-learning platform designed to recruit and train youth to become peer mental health educators by facilitating protective factors to prevent mental health disorders that may lead to youth suicide. The e-learning program openly addresses identified stressors and traumas that put young people at risk for depression, bullying, peer pressure, substance use, and suicide. This is a report on the experiences of a group of participants who pilot-tested the online digital campaign. Participants in this pilot study completed a baseline survey, took part in a self-guided online e-learning course, and were surveyed again postintervention.

## Methods

### Be Present Campaign

**Figure 1:**
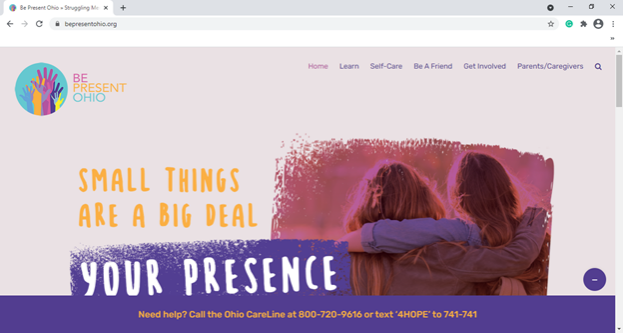
Be Present advocate training online platform.

In October 2017, MEE contracted with the mHealth Impact Lab of the Colorado School of Public Health to evaluate the impact of the e-learning campaign, Be Present, targeting youth suicide prevention in Ohio. Figure 1 displays the home page for the Be Present campaign. MEE trained an inaugural pilot cadre of Be Present Campaign Advocates as peer leaders. mHealth was tasked with evaluating youth (user) engagement with and impacts of the digital e-learning platform. They accomplished this through an assessment of change in self-reported mental health knowledge and protective factors assessed at the start and conclusion of the training.

Here we provide a description of this pilot assessment. This pilot study is classified as a program evaluation (Colorado Multiple Institutional Review Board); therefore, it was not filtered through formal institutional review board processes, although MEE and the mHealth Impact Lab adhered to ethical human subject practices in the execution of the pilot program and evaluation.

### Eligibility Criteria

The Be Present e-learning platform has options for 2 levels of participation open to any Ohio resident aged 14 to 22 years. The first level is called a Be Present Friend, open to those completing the online registration available on the campaign website [12]. After becoming a Friend, youth could participate in the second level and become trainees for Be Present Advocates (ie, peer educators who facilitate youth mental health in their social networks). These Advocates were eligible to complete the comprehensive pretraining and posttraining assessments of this pilot program. The registration process for the Advocate trainees included completing an informed consent or assent form accompanied by parental approval (for youth aged younger than 18 years) and identifying a nonparental adult (meaning not their parent) advisor to support the training process.

### Recruitment

Participants were recruited by MEE primarily through school and community events. Additional recruitment occurred via social and digital media marketing that highlighted the availability of Advocate training. Social media sites such as Facebook, Instagram, Snapchat, and Twitter along with the Be Present website and the campaign’s dedicated YouTube channel were used to recruit youth.

### Data Collection

All data were collected from May 1 to June 1, 2019, by MEE, anonymized, and securely transmitted to mHealth for comprehensive analysis. Several types of data were collected from the Be Present Campaign Advocate training course: (1) data from the pretraining and posttraining surveys (answers to multiple-choice questions) and (2) selected Advocate trainee–entered information in response to questions and assignments in the lessons and modules (answers to multiple-choice and open-ended questions, uploads of assignments, watching instructional videos, etc).

Youth Advocate trainees completed an enrollment survey on the e-learning site before starting the course. After successful enrollment in the Advocate training program, youth watched educational videos and completed online lessons and modules at their own pace. Data on youth engagement with the training curriculum (eg, watching videos, using the safety e-toolbox, completing homework assignments, and sharing messages on social media) were gathered on the training website and maintained by MEE. The social media sites and handles that youth provided during registration were monitored by MEE to determine that assignments requiring a social media share were being completed.

When they were close to completing the online content, Advocate trainees were invited to attend a single community-based training session offered multiple times between May 1 and June 1, 2019, for participant convenience. Attendance was mandatory to receive full Advocate certification. Once Advocate trainees completed the in-person training and while still on-site, they were required to complete a second online survey (posttest) with the same information gathered at baseline. Data from the survey were maintained by mHealth. Following completion of the second survey, trainees were fully certified as Be Present Campaign Advocates.

The pretest and posttest survey, taken anonymously, was developed using Qualtrics. Validated survey questions (Multimedia Appendix 1) were revised from the Rosenberg Self-Esteem Scale [13] and the Community Attitudes Toward Mental Illness questionnaire [14] for measuring mental health stigma, sources of strength, and social support. Additional questions captured standard demographics information, youth resilience measures, positive and negative coping, self-efficacy, and ways to be present for family and friends.

### Measures

Self-report surveys were used to measure 7 youth outcomes: self-efficacy, intentions, behaviors, social support, knowledge, self-esteem, and sources of strength. See Table 1 for a brief description of each youth outcome.

Scores were calculated for each survey domain: self-efficacy, intentions, behaviors, social support, knowledge, self-esteem, and sources of strength. Scores for each survey domain were created by summing scores for each domain question (0=best response, 1=good response, 2=poor response, 3=poorest response). Lower scores indicate better responses.

**Table 1:** Description of youth self-report survey measures.

Outcomes	Description
Self-efficacy	Proportion of respondents who report high measures of their ability to succeed in different situations and tasks. Scored responses report belief and capacity to execute behaviors.
Intentions	Proportion of respondents who report high measures of planned intentions around goals. Scored responses report the aim or plan to execute behaviors.
Behaviors	Proportion of respondents who report referrals to mental health resources for themselves or peers.
Social support	Proportion of respondents who report high measures of social support from family, peers, and community. Scored responses report supportive individuals and community.
Knowledge	Proportion of respondents who report high measures of knowledge around self-care and mental health. Scored responses report knowledge of mental health topics.
Self-esteem	Proportion of respondents with self-satisfaction rated at 3 or more and assessments of feeling no good, useless, or a failure at 2 or fewer on a 5-point scale.
Sources of strength	Proportion of respondents who report high measures of peer social networks. Scored responses report peer social networks.

### Engagement

MEE also captured information from the website portal to the training course. During the self-guided online training for each participant, MEE collected user data that reflects engagement with the training content (ie, lessons and modules, completion of modules) and how that information is internalized by trainees and then shared by them with their adult advisors and peers, either in person or via their existing social media networks. MEE also collected additional data from the Advocate training course, including demographic information on users. A descriptive summary of enrollment, demographic information, and overall user engagement was generated for each completed training module.

### High Risk

We assessed whether youth were at risk themselves for a negative mental health outcome as they responded to questions. These risk-assessing questions were in the behaviors, social support, self-esteem, and sources of strength domains. Participants received an escalated high-risk response message during the survey if they selected disagree or strongly disagree on answers outlined in Multimedia Appendix 1. Each domain had separate criteria; participant had to disagree with all statements within a domain to receive a high-risk message. The high-risk response message was worded as follows:

We hear you. It’s OK to not be OK. We thank you for your openness and honesty in answering these survey questions. Everyone has a bad day from time to time. But sometimes, something more might be going on. If you’re feeling hopeless, overwhelmed, or in a crisis, it might be time to get some immediate support to help you get through it. When the emotional pain seems too big to handle, get help. Start with these resources where people will step up and Be Present for you... To talk to someone, text 4HOPE to the Crisis Text Line (741741) or call the National Suicide Prevention Lifeline (1-800-273-8255). Both are available 24/7.

The algorithm and high-risk response messages were vetted by clinical and research experts. Each respondent could receive up to 4 escalated high-risk response messages per domain.

### Analysis

All statistical analysis was performed using SAS 9.4 (SAS Institute Inc). The study data used in this analysis were collected by MEE team members and managed using Qualtrics survey software hosted at the University of Colorado Anschutz Medical Campus, where mHealth resides. All data were anonymized and reviewed for completeness and consistency. Descriptive statistics were performed to understand the study population, summarize enrollment and engagement, and detect any erroneous values.

A descriptive assessment was performed integrating Be Present e-learning engagement data from MEE with single-arm, pretest, and posttest assessments with Be Present Advocates. The Student *t* test was used to assess differences in mean scores on each of the survey domains between pretest and posttest. *P*<.05 was considered to be statistically significant. Engagement with the digital e-learning platform was measured as the percentage of participants who completed each of the training blocks. Changes in self-reported intentions, self-efficacy, and behaviors related to mental health were measured at baseline and postparticipation in the Be Present Campaign in a single-arm design. The study enrolled 65 participants, of which 62 had data from the baseline survey and 54 had data from both baseline and follow-up surveys. Only participants who had baseline data were included in the analysis.

High-risk responses were also analyzed using descriptive analytics to determine the characteristics of respondents who received escalated high-risk messages during the survey completion. These were survey questions identifying high-risk respondents. High-risk messages were positioned in the domains behaviors, social support, self-esteem, and sources of strength.

## Results

### Demographics

Table 2 reports the characteristics of Be Present Campaign Advocate trainees who completed the pretraining (baseline) survey. Of the respondents, 82% (51/62) identified as female and 95% (59/62) as straight or heterosexual. A total of 77% (48/62) of respondents were non-Hispanic White, 10% (6/62) identified as Hispanic, and 13% (8/62) as non-Hispanic non-White.

**Table 2:** Demographic characteristics of participants in the Be Present online training intervention pilot test for youth suicide prevention at baseline, May-June 2019 (n=62)^a^.

Characteristic			Value, n (%)
**Gender**			
	Male	11 (18)	
	Female	51 (82)	
	Other	—^b^	
	Prefer not to answer	—	
**Sexual orientation**			
	Straight or heterosexual	59 (95)	
	Lesbian, gay, or homosexual	1 (2)	
	Bisexual	1 (2)	
	Prefer not to answer	1 (2)	
**Race/ethnicity**			
	Hispanic or Latino	6 (10)	
	Non-Hispanic White	48 (77)	
	Non-Hispanic non-White	8 (13)	

^a^Percentages may not add up to 100 due to rounding.

^b^Not applicable.

### Engagement

This is a descriptive summary of user engagement in the Be Present online training pilot test for youth suicide prevention, May-June 2019. Of the participants enrolled, 60% (37/62) completed all 7 training blocks.

The completion percentages for each training block were as follows: self-efficacy (54/62, 87%), intentions (53/62, 85%), behaviors (51/62, 82%), social support (45/62, 73%), knowledge (37/62, 60%), self-esteem (37/62, 60%), and sources of strength (37/62, 60%).

### Youth Behavioral Outcomes

Table 3 illustrates the percentage improvement in scores for each survey domain. There were statistically significant improvements in behaviors (2.75, *P*=.007) and social support (4.13, *P*<.001). All other categories saw modest improvement from pretest to posttest.

**Table 3:** Changes in domain scores between baseline and postintervention surveys.

Domain	Pretest, mean	Posttest, mean	Percentage change in score	*P* value
Self-efficacy	13.50	14.31	0.81	.42
Intentions	7.38	7.60	0.22	.83
Behaviors	31.85	34.60	2.75	.007
Social support	62.67	66.80	4.13	.001
Knowledge	24.98	25.98	1.00	.32
Self-esteem	21.48	21.78	0.30	.76
Sources of strength	23.04	24.93	1.89	.06

### High-Risk Responses

Table 4 outlines the proportion of participants who selected answers that prompted an escalated high-risk response message. Of the respondents who completed the pretest, 15% (9/62) of respondents received a total of 13 escalated high-risk response messages. Five respondents received an escalated high-risk response message, triggered when they indicated engaging in risky behaviors or having limited social support, self-esteem, or sources of strength. Each respondent could receive up to 4 escalated high-risk messages. The remaining survey domains do not have questions identifying high-risk respondents. The most escalated response messages were to females across the survey domains social support (n=5), self-esteem (n=6), and sources of strength (n=1). Among respondents who completed both the pretest and posttest survey, 9% (5/54) received a total of 7 escalated high-risk messages in the posttest survey. Females received the most escalated response messages across the survey domains: behaviors (n=2) and self-esteem (n=4).

**Table 4:** High-risk responses by category, pretest and posttest.

Domain	Pretest		Posttest		Total
	Male, n (%)	Female, n (%)	Male, n (%)	Female, n (%)	
Behaviors	—^a^	—	—	2 (33)	2
Social support	1 (1)	5 (42)	—	—	6
Self-esteem	—	6 (50)	1 (1)	4 (67)	11
Sources of strength	—	1 (8)	—	—	1
Total	1 (5)	12 (60)	1 (5)	6 (30)	20

^a^Not applicable.

## Discussion

### Principal Findings

The objective of this study was to present descriptive results of a primarily digital pilot mental health training program for Ohio youth. For this paper, we focused specifically on the impact on youth behavioral outcomes, user engagement, and identifying those at high-risk for immediate intervention. Our results are process-focused by design as a way of providing information to improve the overall program and implementation.

### Youth Behavioral Outcomes

Youth behavioral outcomes were assessed immediately posttraining, which reflects an immediate short-term outcome rather than a sustained immediate or longer term outcome. We did observe a significant increase in self-report of referrals for mental health services as well as in perceptions that youth had of experiencing social support. These results offer some initial optimism considering that the Be Present program may have potential for impact, although we cannot suggest this is a definitive conclusion without a larger and more rigorous examination of the program.

In the other domains explored, including intentions, self-efficacy, knowledge, and sources of strength, we observed no significant difference between pretest and posttest self-assessments for youth. Given that we have a small sample and no control group, our findings should be considered only preliminary to a larger trial and underscore primarily the feasibility of delivering online training programs to bolster youth mental health.

### Engagement

Close to two-thirds of the enrollees completed all of the Advocate training modules. The first 3 training blocks (blocks 1, 2, and 3) had a higher percentage of enrollee completion compared with the latter 3 blocks (blocks 4, 5, and 6). A systematic review of mobile and web-based interventions indicated that engagement decreases in interventions that have a longer duration [15]. Although we are uncertain as to why there was a gradual decline in engagement, we speculate that intervention duration may have played a role. Also, enrollees were allowed to complete the modules at their own pace, which may have negatively impacted sustained interest and engagement. A solution might include reducing the number of training modules and setting a time limit for module completion.

### High-Risk Responses

Most respondents who received escalated high-risk response messages identified as female. Due to the small number of male respondents, this may not be representative of the larger population. However, evidence has shown that adolescent and adult females report a higher percentage of suicidal ideations compared with their male counterparts [16,17]. A full-scale intervention evaluation examining high-risk responses is required to suggest gender differences among Ohio adolescents.

### Limitations and Strengths

Although we discovered interesting descriptive findings that will inform modifications to the next iteration of Be Present, the overall study is not without limitations. The majority of respondents in this pilot identified as white, female, and straight or heterosexual. While reflective of the larger school population, this is not generalizable. Only 60% of enrollees completed the whole training with no indication as to why other enrollees did not complete the training. This introduces the potential for biased results as we do not know the impetus for or against completing the training.

Self-report surveys yielded questionable responses as respondents answered every question identically in several cases, raising concern with the study’s external validity. This is a limitation of the pretest and posttest evaluation. However, exploration of ways to improve the accuracy of self-report among adolescents may be helpful.

We also recognize process-related limitations. The survey should have been streamlined to include one link to improve the delivery of questions, reduce data processing requirements, and avoid distribution issues. Not enough time was allotted between the training and surveys to draw more conclusive results on the training’s impact. Although we can describe engagement with Advocate training data, we cannot make conclusions on effectiveness. Future steps should include the possibility of evaluating effectiveness and efficacy, similar to other digital mental health pilot programs [18].

One of several strengths of the study is that it provides insights into future implementation for the Be Present program. These data will be used to modify the training program and implementation to improve both process and engagement and will be useful for understanding the impact of mental health advocacy at certain points in youth development. Finally, this study has implications for the future of youth mental wellness social media campaigns and youth engagement.

### Future Work

We plan to implement the Be Present program in other areas using the insights gained from this pilot. Next steps in the Be Present program include the addition of more diverse populations to evaluate behavioral outcomes and engagement. Furthermore, a more streamlined process will allow for more time between Advocate training and outcome measures. Our goal is to test for the preliminary efficacy of the program. Follow-up studies from this pilot will be robust enough to allow for more complex statistical approaches to truly capture changes in self-report attitudes toward mental health and determine if engagement with the campaign is associated with youth behavioral outcomes. Although this analysis cannot be used to make full conclusions on the impact on youth behavioral outcomes, it does give key insight into the impact of digital training platforms.

### Conclusion

In this paper, we provided descriptive analyses for a peer advocate mental health digital intervention program for youth in Ohio. The important take-away from this study is that it takes time to develop a solid digital mental health program, especially for adolescents. Those who venture into developing prevention programs must be prepared for more than one iteration of a pilot. Factors beyond what is discussed in this article confound the process for a seamless implementation—duration uncertainties, relatability to the content, range in developmental maturity, access to the internet and technology devices, and other competing factors like schoolwork and family obligations. Although the internet and ready availability of devices make programs more accessible, adolescents are typically still at the mercy of a parent who can determine if and when their child can use the internet and devices. For this reason, it is beneficial for prevention intervention programs to include a formative process evaluation in which one can monitor engagement and implementation and make the necessary changes as they go. Program development is time-consuming and expensive, and thus modifying as one goes is prudent.

## Appendix

Multimedia Appendix 1 High-risk assessment.

## Abbreviations

MEE: Motivational Educational Entertainment Productions Inc
MHAS: Ohio Department of Mental Health and Addiction Services
