# Supplementary material for: Implementing a Peer Advocate Mental Health Digital Intervention Program for Ohio Youth: Descriptive Pilot Study
Source: JMIR Ment Health. 2021 Apr 23;8(4):e24605. doi: 10.2196/24605 (PMC8105753; doi:10.2196/24605)
Supplement: Multimedia Appendix 1 [file mental_v8i4e24605_app1.docx]

Block: Behaviors

*I’ve been using alcohol or drugs to make myself feel better and/or to help me get through my situation* – Strongly agree or Agree is selected

*I’ve given up trying to deal with unpleasant situations in my life –* Strongly agree or Agree is selected

*I’ve given up attempting to cope with my situation –* Strongly agree or Agree is selected

Block: Social Support

*I talk to my family/caregiver(s) about how I feel –* Strongly Disagree or Disagree is selected

*I feel supported by my friends –* Strongly Disagree or Disagree is selected

*My family stands by me during difficult times –* Strongly Disagree or Disagree is selected

*My friends stand by me during difficult times –* Strongly Disagree or Disagree is selected

*I feel safe when I am with my family/caregiver(s) –* Strongly Disagree or Disagree is selected

Block: Self Esteem

*As a whole, I am satisfied with myself –* Strongly Disagree or Disagree is selected

*At times, I think I am no good at all –* Strongly Agree or Agree is selected

*I certainly feel useless at times –* Strongly Agree or Agree is selected

*All in all, I am inclined to feel that I am a failure –* Strongly Agree or Agree is selected

Block: Sources of Strength

*I believe I can find a purpose in life, a reason to live –* Strongly Disagree or Disagree is selected

*I believe I can learn to adjust or cope with my problems –* Strongly Disagree or Disagree is selected

*I have future plans I am looking forward to carrying out –* Strongly Disagree or Disagree is selected
